# Chemical Modification of Cysteine with 3-Arylpropriolonitrile Improves the In Vivo Stability of Albumin-Conjugated Urate Oxidase Therapeutic Protein

**DOI:** 10.3390/biomedicines9101334

**Published:** 2021-09-27

**Authors:** Byungseop Yang, Inchan Kwon

**Affiliations:** School of Materials Science and Engineering, Gwangju Institute of Science and Technology (GIST), Gwangju 61005, Korea; yangbs@gm.gist.ac.kr

**Keywords:** thiol-maleimide, 3-arylpropiolonitriles, half-life extension, site-specific albumin conjugation, urate oxidase, therapeutic protein

## Abstract

3-arylpropiolonitriles (APN) are promising alternatives to maleimide for chemo-selective thiol conjugation, because the reaction product has a remarkably hydrolytic stability compared with that of thiol-maleimide reactions in vitro. However, whether cysteine modification with APN enhances stability in vivo compared to thiol-maleimide reactions remains unclear, probably due to the too short in vivo serum half-life of a protein to observe significant cleavage of thiol-maleimide/-APN reaction products. The conjugation of human serum albumin (HSA) to a therapeutic protein reportedly prolongs the in vivo serum half-life. To evaluate the in vivo stability of the thiol-APN reaction product, we prepared HSA-conjugated *Arthrobacter globiformis* urate oxidase (AgUox), a therapeutic protein for gout treatment. Site-specific HSA conjugation to AgUox was achieved by combining site-specific incorporation of tetrazine containing an amino acid (frTet) into AgUox and a crosslinker containing trans-cyclooctene and either thiol-maleimide (AgUox-MAL-HSA) or -APN chemistry (AgUox-APN-HSA). Substantial cleavage of the thioester of AgUox-MAL-HSA was observed in vitro, whereas no cleavage of the thiol-APN product of AgUox-APN-HSA was observed. Furthermore, the in vivo serum half-life of AgUox-APN-HSA in the late phase was significantly longer than that of AgUox-MAL-HSA. Overall, these results demonstrate that the thiol-APN chemistry enhanced the in vivo stability of the HSA-conjugated therapeutic protein.

## 1. Introduction

The thiol group (-SH) has been widely used for the chemical modification of biomolecules [1,2,3,4,5]. In particular, because cysteine residues of proteins are relatively rarer than other reactive amino acids, such as lysines, they are often used for site-specific modifications [6,7,8,9,10]. Maleimides are widely used as the reaction partners of cysteines of proteins [11,12,13,14]. The thiol-maleimide reaction generates a thioether bond at a pH range of 6.5–7.5 and ensures realization of the desired conjugates with a high yield [11,12,13,14]. Given its selectivity, efficiency, and accessibility under moderate reaction conditions, thiol-maleimide chemistry has been used for the development of a variety of materials ranging from chemical drugs to therapeutics for several decades [11,12,13,14]. However, thiol-maleimides also generate the thioether group, which is unstable over a long period in aqueous media and in vivo conditions, and undergoes retro-conjugate additions and subsequent trapping with endogenous thiols, such as glutathione in vivo [15,16,17,18,19]. This major drawback of thiol-maleimide chemistry hampers the in vivo application of protein conjugates, such as antibody–drug conjugates [20,21,22]. To overcome this limitation, 3-arylpropiolonitriles (APN) were developed as alternatives to maleimides [19,23,24]. The thiol-APN chemistry is highly chemo-selective in aqueous buffer conditions [19]. In addition, the product of the thiol-APN reaction is more stable than that of the thiol-maleimide reaction in vitro [19]. Considering the significant potential of thiol-APN reactions in various in vivo applications, it is important to evaluate the in vivo stability of the thiol-APN reaction product of a protein. However, to our knowledge, the in vivo stability of thiol-maleimide and thiol-APN reaction products of proteins has not yet been compared. We speculated that the serum half-lives of most proteins are too short to observe the cleavage of the thiol-maleimide/-APN reaction product in vivo. To circumvent this limitation, proteins with a sufficiently long serum half-life are required.

In this study, to evaluate the in vivo stability of the thiol-APN reaction product, we investigated the in vivo stability of a human serum albumin (HSA)-therapeutic protein conjugate. Conjugation of HSA to therapeutic agents, including chemical drugs and therapeutic proteins, is an effective strategy to extend the serum half-life [25,26,27,28]. HSA has an exceptionally long serum half-life in humans (approximately 3 weeks) due to the neonatal Fc receptor (FcRn)-mediated recycling mechanism [29,30]. There is only one free cysteine at position 34 (Cys34) on the HSA surface, which is far from the FcRn binding domain [29,30]. Therefore, the Cyst34 of HSA allows the conjugation of fluorescence labeling dyes, chemical drugs, or therapeutic proteins using the thiol-maleimide reaction without perturbing the FcRn binding [27,28,29,30]. We previously reported that site-specific albumin conjugation to the permissive site of a protein results in in vivo serum half-life extension without loss of activity [30,31].

**Figure 1:**
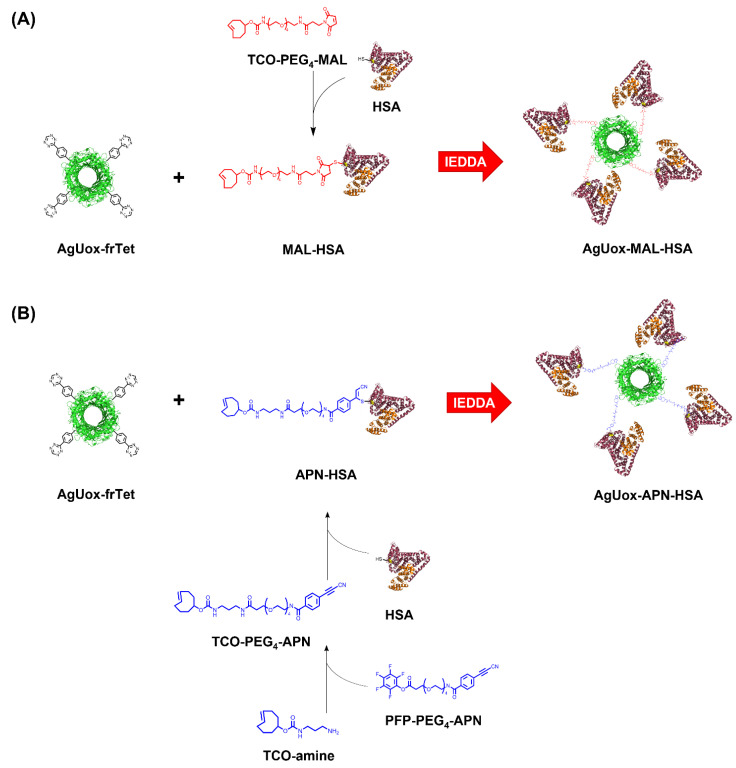
Schematic representation of site-specific human serum albumin conjugation to AgUox-frTet to prepare AgUox-MAL-HSA (**A**) and AgUox-APN-HSA (**B**). AgUox-frTet: AgUox containing frTet; MAL-HSA: HSA conjugated to TCO-PEG_4_-MAL linker via thiol-maleimide reaction; TCO-PEG_4_-APN: TCO-amine reacted with PFP-PEG_4_-APN; APN-HSA: HSA conjugated to TCO-PEG_4_-APN linker via thiol-APN reaction.

To compare the in vivo stabilities of thiol-maleimide and thiol-APN reaction products, we first site-specifically incorporated a phenylalanine containing a fast-reacting tetrazine (frTet) into urate oxidase (Uox) using the optimized *Methanococcus jasnacci* tyrosyl-tRNA/synthetase (MjtRNA^Tyr^/MjTyrRS) pair [32]. The recombinant Uox and poly-ethylene glycol-conjugated Uox are therapeutic proteins to treat tumor lysis syndrome and gout, respectively [33,34,35]. We chose Uox as a model therapeutic protein, since it is convenient to produce from *E. coli* expression hosts, and its activity can be determined by a simple biochemical assay. In previous studies, the urate oxidase derived from *Aspergillus flavus* (AfUox) was used [30,36]. However, since AfUox has a low thermostability, it is not ideal to evaluate the in vivo stability of thiol-APN reaction products over a long period [37]. Therefore, we chose a thermostable Uox derived from *Arthrobacter globiformis* (AgUox) in this study [30,36,38,39]. Then, a hetero-bifunctional crosslinker containing a trans-cylooctene (TCO) group and either maleimide or APN was conjugated to the Cys34 of HSA to prepare MAL-HSA or APN-HSA, respectively (Figure 1). Next, the AgUox variant containing frTet (AgUox-frTet) was conjugated to MAL-HSA or APN-HSA to generate a AgUox-MAL-HSA or AgUox-APN-HSA conjugate via inverse electron demand Diels-Alder reaction, respectively (Figure 1). We evaluated the in vitro and in vivo stabilities of AgUox-MAL-HSA and AgUox-APN-HSA. The number of HSA molecules conjugated to a therapeutic protein is reportedly directly correlated to the in vivo serum half-life [40]. Therefore, the cleavage of a thiol reaction product will release HSA from AgUox-HSA conjugates, resulting in a shortened in vivo serum half-life. Therefore, as a measure of the in vivo stability of AgUox-HSA conjugates, the in vivo serum half-lives of the conjugates were compared.

## 2. Materials and Methods

### 2.1. Materials

frTet (4-(1,2,3,4-tetrazin-3-yl) phenylalanine) was purchased from Aldlab Chemicals (Woburn, MA, USA). TCO-Cy3 was purchased from AAT Bioquest (Sunnyvale, CA, USA). TCO-PEG4-maleimide (TCO-PEG_4_-MAL) and amine-axially substituted TCO (TCO-amine) were purchased from FutureChem (Seoul, Korea). Pentafluorophenyl ester (PFP)-PEG_4_-APN was obtained from CONJU-PROBE (San Diego, CA, USA). Disposable PD-10 desalting columns and Superdex 200 10/300 GL Increase columns were purchased from Cytiva (Uppsala, Sweden). Vivaspin 6 centrifugal concentrators with a molecular weight cut-off (MWCO) of 10 and 100 kDa were purchased from Sartorius (Göttingen, Germany). HSA and all other chemical reagents were purchased from Sigma-Aldrich (St. Louis, MO, USA), unless otherwise mentioned.

### 2.2. Generation of pBAD_AgUox and pBAD_AgUox-196Amb Plasmids for Expression of AgUox Variants (Wild-Type AgUox and AgUox-frTet)

The gene encoding AgUox was synthesized by Macrogen (Seoul, South Korea). To express wild-type AgUox (AgUox-WT) or AgUox-frTet, the synthesized gene was used as a template and amplified using polymerase chain reaction (PCR), using the specific primers pBAD-AgUox_F (5′-GCCGCCATGGTGTCTGCTGTGAAGG-3′) and pBAD-AgUox_R (5′-GCCGAGATCTTTAATGGTGATGGTG-3′). The amplified gene was digested with two restriction enzymes (*Nco*I and *Bgl*II), and inserted between the *Nco*I and *Bgl*II sites of the pBAD vector to generate pBAD_AgUox. To replace the glutamic acid codon at position 196 with an amber codon (UAG), site-directed mutagenesis PCR was performed using pBAD-AgUox as a template and the primers AgUox-196Amb_F (5′-GTCGAAGTCCACCTATACGGTGTTGTAACGCCAACGG-3′) and AgUox-196Amb_R (5′-CCGTTGGCGTTACAACACCGTATAGGTGGACTTCGAC-3′) to generate pBAD-AgUox_196amb.

### 2.3. Expression and Purification of AgUox-WT and AgUox-frTet

To express AgUox-frTet, C321delA.exp [pDule_C11] [pBAD_AgUox-196Amb] *Escherichia coli* cells containing the optimized MjtRNA^Tyr^/MjTyrRS toward frTet were prepared and used as previously reported [36]. *E. coli* cells cultured in Luria broth (LB) medium containing ampicillin (100 μg/mL) and tetracycline (10 μg/mL) at 37 °C overnight with shaking were inoculated into 2× YT medium containing conditions identical to those of the LB medium. After 2.5 h of shaking incubation, frTet and *L*-(+)-arabinose were added to the medium to final concentrations of 1 mM and 0.4% (*w/v*), respectively, when the optical density (600 nm) of the medium reached 0.5. After incubation for 5 h, the cells were harvested by centrifugation at 5000 rpm for 10 min at 4 °C. Purification of AgUox-frTet was performed by immobilized-metal affinity chromatography using polypropylene columns packed with nickel-nitrilotriacetic acid (Ni-NTA) agarose resins at 4 °C according to the manufacturer’s instructions (Qiagen). Purified proteins were desalted with PBS (pH 7.4) using PD-10 columns. The expression and purification processes of the AgUox-WT were similar to those of AgUox-frTet, but without the addition of tetracycline and frTet in the culture medium during the expression step. The cultured cells and purified AgUox variants were subjected to SDS-PAGE analyses using tris-glycine gels (5% acrylamide stacking and 12 % acrylamide resolving gels) run at 120 V in the running buffer (25 mM Tris, 192 mM glycine, and 0.1% SDS at pH 8.3). The molecular weight standard marker (Bio-Rad Laboratories Inc., Berkeley, CA, USA) was used.

### 2.4. MALDI-TOF Mass Spectrometry (MS) Analysis

First, either AgUox-WT or AgUox-frTet was digested with trypsin according to the manufacturer’s protocol. A total of 0.4 mg/mL of Uox variants (AgUox-WT and AgUox-frTet) was digested overnight at 37 °C, and then desalted using ZipTip C18. Tryptic digested mixtures were mixed with a 2,5-dihydroxybenozic acid (DHB) matrix solution (20 mg/mL of DHB in 30:70 (*v/v*) acetonitrile: trifluoroacetic acid 0.1% in water) and then analyzed using a Microflex MALDI-TOF/MS device (Bruker Corporation, Billerica, MA, USA).

### 2.5. Site-Specific Fluorescence Dye Labeling of AgUox-WT and AgUox-frTet

Purified AgUox-WT and AgUox-frTet were reacted with TCO-Cy3 fluorescence dye in a 1:2 molar ratio at room temperature in PBS (pH 7.4). After 2 h, the reaction mixtures were subjected to sodium dodecyl sulfate polyacrylamide gel electrophoresis (SDS-PAGE). The fluorescence image of the protein gel was obtained using a ChemiDoc XRS+ system (illumination at 302 nm, filter from 510–610 nm; Bio-Rad Laboratories, Hercules, CA, USA). After fluorescence analysis, the protein gel was stained with Coomassie Brilliant Blue R-250 dye. The protein gel image was obtained using the ChemiDoc XRS+ system using white-light illumination.

### 2.6. Generation of AgUox-HSA Conjugates (AgUox-MAL-HSA and AgUox-APN-HSA)

To perform site-specific albumination into AgUox, HSA was purified via anion exchange chromatography using an HiTrap Q HP anion exchange column as previously reported [30,31]. The purified HSA was desalted with PBS (pH 7.0) and then reacted with TCO-MAL at a molar ratio of 1:4 in PBS (pH 7.0) at room temperature. After 2 h, the reaction mixture was desalted with PBS (pH 7.4) using a PD-10 column to remove the unreacted TCO-PEG_4_-MAL linker to obtain the MAL-HSA conjugate. The purified Uox-frTet was reacted with MAL-HSA at a molar ratio of 1:4 in PBS (pH 7.4) at room temperature for 5 h. After conjugation, the reaction mixture was subjected to size exclusion chromatography (SEC) (Superdex 200 increase 10/300 column, 0.25 mL/min flow rate, and absorbance measurement at 280 nm) using the NGC Quest 10 Plus Chromatography System (Bio-Rad Laboratories Inc., Berkeley, CA, USA). The molecular weight and purity of the eluted fractions were analyzed using SDS-PAGE, and the fraction corresponding to AgUox-frTet conjugated to four MAL-HSA molecules (AgUox-MAL-HSA) was selected and concentrated for further analysis.

To generate the AgUox-HSA conjugate via a hetero-bifunctional cross-linker containing TCO and APN, TCO-amine was reacted with PFP-PEG_4_-APN at a molar ratio of 1:1 in DMSO at room temperature for 30 min. The purified HSA was buffer-exchanged to 50 mM sodium borate buffer (pH 9.0) using a PD-10 desalting column. The purified HSA was reacted with TCO-PEG_4_-APN at a molar ratio of 1:4 in 50 mM sodium borate buffer (pH 9.0) at room temperature for 2 h. To remove the unreacted TCO-APN linker, the reaction mixture was desalted with PBS (pH 7.4) using a PD-10 column. The conjugation and purification of AgUox-frTet conjugated to four HSA molecules via a linker containing APN (AgUox-APN-HSA) were performed in a similar manner to those of AgUox-MAL-HSA.

### 2.7. Enzymatic Activity and Stability Assays of AgUox-WT and AgUox-HSA Conjugates (AgUox-MAL-HSA and AgUox-APN-HSA)

Prior to the enzymatic activity assays of AgUox-WT and AgUox-HSA conjugates (AgUox-MAL-HSA and AgUox-APN-HSA), the protein concentrations were determined by the BCA method (Thermo Scientific, Wilmington, DE, USA). Uric acid-degrading enzymatic activity of AgUox was measured spectrophotometrically as previously described [30]. Briefly, 100 μL of 200 μM uric acid in enzymatic activity assay buffer (50 mM sodium borate and 0.2 M NaCl, pH 9.5) was added to 100 μL of 120 nM purified AgUox-WT or AgUox-HSA conjugate. The uric acid-degrading enzymatic activity was determined by absorbance change at 293 nm. The serum activity of AgUox variants was measured by adding 100 μL of enzyme activity assay buffer containing 100 μM of uric acid into 100 μL enzyme activity buffer containing 5 μL of serum, and then measured as described above.

The AgUox-HSA conjugates were incubated at 37 °C in PBS buffer (pH 7.4) containing 5 μM glutathione and 20 μM HSA, mimicking normal blood conditions, for five days [17]. Then, the samples were analyzed by SDS-PAGE.

### 2.8. In Vivo Stability Assays of AgUox Variants

The stability assays of AgUox variants in mice were performed according to the guidelines of the Animal Care and Use Committee of the Gwangju Institute of Science and Technology (GIST-2020-037). AgUox-WT, AgUox-MAL-HSA, or AgUox-APN-HSA (5.0 nmol based on AgUox monomer in 200 μL PBS at pH 7.4) was injected into the tail vein of young female BALB/c mice (*n* = 4). The blood samples were collected via retro-orbital bleeding at 15 min, 3, 6, and 12 h for AgUox-WT, or at 15 min, 12, 24, 48, 72, 84, 96, 108, and 120 h for the AgUox-HSA conjugates. After separation of serum from the blood, the serum activity of AgUox variants was determined using the method described above.

## 3. Results and Discussion

### 3.1. Site-Specific Incorporation of frTet into AgUox

To investigate whether the thiol-APN reaction product is more stable than that of thiol-MAL in vivo, we prepared two HSA-conjugated therapeutic protein variants. As a model therapeutic protein, we chose AgUox, a homotetrameric enzyme consisting of identical 34 kDa subunits with high thermostability and high expression level in *E. coli*, and thus has great potential to treat hyperuricemia conditions/gout [30]. First, we prepared AgUox containing frTet (Figure 1) by adapting expression and purification methods previously described except that AgUox was used instead of *Aspergillus flavus* Uox [36]. Among the non-natural amino acids containing the tetrazine group for the IEDDA reaction, frTet, whose reactivity has been well defined [32,36,40], was successfully incorporated into the protein.

**Figure 2:**
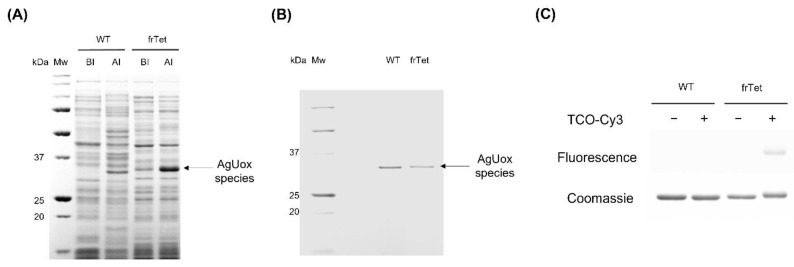
SDS-PAGE analysis of AgUox variants (AgUox-WT and AgUox-frTet). (**A**) Coomassie Brilliant Blue (CBB)-stained protein of AgUox variants. Lanes: Mw, molecular weight marker; BI, before induction; AI, after induction. (**B**) CBB-stained protein gel of purified AgUox variants. (**C**) Fluorescence image and CBB-stained protein gels of AgUox variants incubated in the absence (–) or presence (+) of TCO-Cy3.

We then carefully selected the frTet incorporation site of AgUox, which is a critical step, as it impacts the thermodynamic folding and three-dimensional structure of the protein. The AgUox variant containing frTet at position 196 exhibited good thermostability and enzymatic activity comparable to those of wild-type AgUox [36]. To site-specifically incorporate frTet at position 196 of AgUox (AgUox-frTet), we prepared *E. coli* co-transformed with the plasmid encoding the engineered orthogonal pair of MjtRNA^Tyr^/MjTyrRS and pBAD-AgUox_196Amb. We successfully expressed AgUox variants (AgUox-WT and AgUox-frTet) and purified them using IMAC purification. We then compared the cell lysate before and after induction using SDS-PAGE and subsequent Coomassie blue staining. A protein band with a molecular weight of approximately 34 kDa corresponding to the AgUox monomer was observed after induction, confirming that the protein was expressed (Figure 2A). The observation of Uox monomers, instead of the tretrameric complex, could be explained by the disruption of the non-covalent bonds of the homotetrameric Uox by SDS treatment. The Coomassie blue-stained gel of the purified AgUox variants indicated that the molecular weight of AgUox-WT and AgUox-frTet was approximately 34 kDa (Figure 2B), in line with previous reports [30,40]. The yields of purified AgUox-WT and AgUox-frTet were 40.1 and 19.2 mg/L, respectively.

### 3.2. Confirmation of Site-Specific Incorporation of frTet into AgUox Using Fluorescence Dye Labeling and MALDI-TOF MS

**Figure 3:**
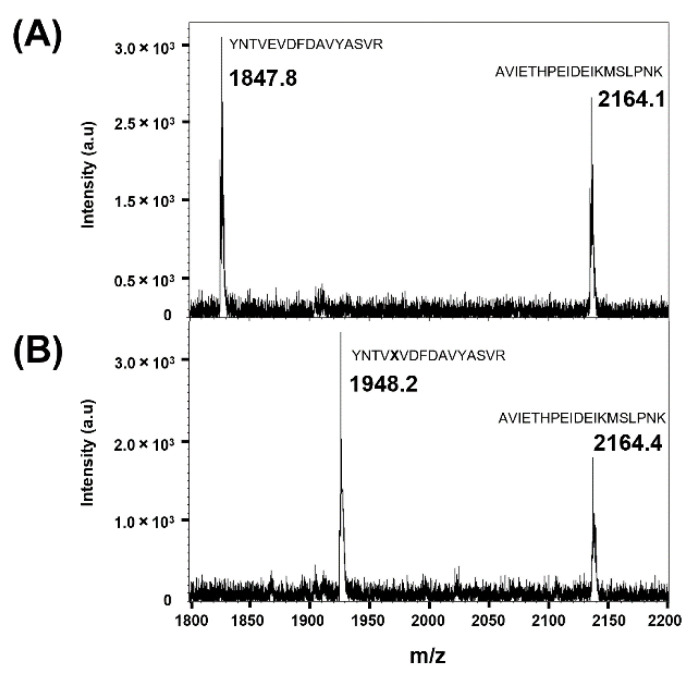
Matrix-assisted laser desorption/ionization-time of flight mass spectra of trypsin-digested fragments of AgUox-WT (**A**) and AgUox-frTet (**B**). The mass of the AgUox-WT fragment, YNTVEVDFDAVYASVR (residues 192–207), was compared to that of the AgUox-frTet fragment, YNTVXVDFDAVYASVR, where X indicates frTet. The peak of fragment AVIETPEIDEIKMSLPNK (residues 232–250) was used as a control.

To confirm whether frTet was incorporated at position 196 of AgUox, trypsin-digested fragments of AgUox-WT and AgUox-frTet were subjected to MALDI-TOF MS. In the spectra of tryptic digests of AgUox samples, the peak representing the AVIETHPEIDEIKMSLPNK peptide of AgUox (positions 232–250) had a mass of 2164.1 m/z, corresponding to the expected one (Figure 3). The trypsin-digested fragment of AgUox-WT containing E196 had a mass of 1847.8 m/z (Figure 3), which matches well with the expected mass of the YNTVEDFDAVYASVR peptide (positions 192–207) of AgUox (1847.9 m/z). Replacing glutamic acid at position 196 (E196) with frTet (245.2 Da) was expected to shift the m/z from 1847.9 to 1948.0 m/z. In the spectra of trypsin-digested AgUox-frTet, a new peak was detected at 1948.2 m/z (Figure 3), clearly showing that E196 of AgUox was substituted by frTet.

Next, fluorescence dye labeling was performed to investigate whether the frTet in AgUox exhibited IEDDA reactivity. The purified AgUox-WT and AgUox-frTet were reacted with TCO-Cy3. AgUox-WT was used as a negative control. As expected, no band for AgUox-WT was observed in the fluorescence image of the protein gel, regardless of the reaction with TCO-Cy3 (Figure 2C, fluorescence panel). In the case of AgUox-frTet, the protein band was observed in the fluorescence image of the protein gel, only when the sample was reacted with TCO-Cy3 (Figure 2C, fluorescence panel). After Coomassie Brilliant Blue staining of the protein gel, the bands of all proteins regardless of reaction with TCO-Cy3 were observed (Figure 2C, Coomassie panel). Overall, the results of fluorescence dye labeling demonstrate that frTet incorporated into AgUox retained its reactivity to IEDDA.

### 3.3. Site-Specific HSA Conjugation to AgUox and Purification of AgUox-HSA Conjugates

**Figure 4:**
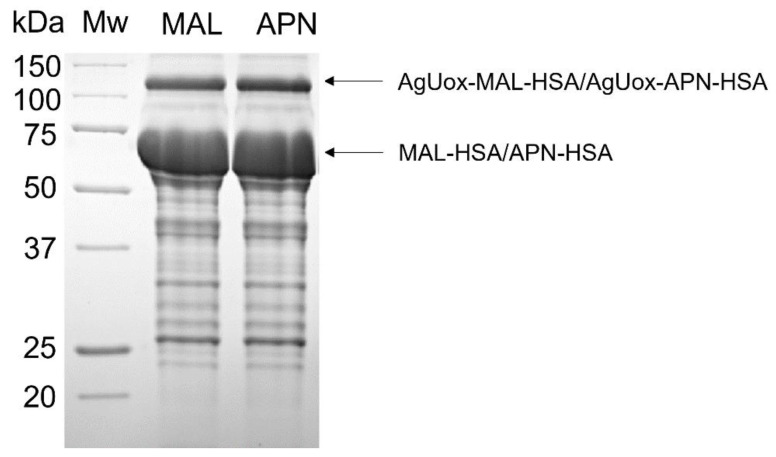
Protein gel of the reaction mixture of AgUox-frTet with either MAL-HSA (MAL lane) or APN-HSA (APN lane). Mw: molecular weight standards.

**Figure 5:**
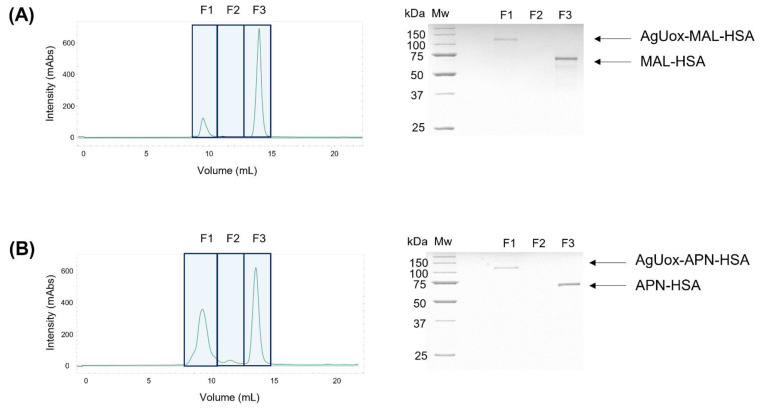
Size exclusion chromatograms and the protein gel of fractions of the conjugation mixture of AgUox-frTet and either MAL-HSA (**A**) or APN-HSA (**B**). Eluted fractions were loaded onto a protein gel and then stained with Coomassie Brilliant Blue.

To prepare the AgUox-MAL-HSA conjugate, two-step conjugation was performed using TCO-PEG_4_-MAL as the crosslinker agent (Figure 1). First, the TCO-PEG_4_-MAL crosslinker was conjugated to HSA to generate MAL-HSA. Specifically, the thiol group of the cysteine at position 34 (C34) of HSA reacted with maleimide via the Michael addition. The C34 position has been widely used for site-specific albumin conjugation to enhance the serum half-life of proteins in vivo because it is far from the FcRn binding site and has high solvent accessibility [26,30]. Second, the MAL-HSA conjugate was reacted with AgUox-frTet via IEDDA to generate an AgUox-MAL-HSA conjugate containing a thioether group. SDS-PAGE analysis of the reaction mixture indicated that the conjugation yield of AgUox-HSA was greater than 90% (Figure 4). After the IEDDA reaction, SEC was applied to separate AgUox-MAL-HSA from the conjugation mixture. Two main peaks were detected in the chromatogram containing F1 and F2 fractions (Figure 5A). SDS-PAGE analysis of all fractions indicated that the F1 and F2 fractions contained AgUox-MAL-HSA conjugate and unreacted MAL-HSA, respectively (Figure 5A). These results demonstrate the successful site-specific conjugation of HSA to AgUox via IEDDA and Michael addition. The yields of the AgUox-HSA conjugate variants (AgUox-MAL-HSA and AgUox-APN-HSA) produced from AgUox-frTet were 6.1 and 9.2 mg/L, respectively.

To generate AgUox-APN-HSA, three-step conjugation was performed. First, we synthesized a TCO-PEG_4_-APN crosslinker via a reaction between TCO-amine and PFP-PEG_4_-APN. Although the chemical properties of PFP ester are similar to those of *N*-hydroxysuccinimide ester, it is more reactive with amine groups [37,41]. After coupling TCO-amine with the PFP-APN linker, the methods for the conjugation and purification of AgUox-APN-HSA were similar to those of AgUox-MAL-HSA, except that the TCO-PEG_4_-APN crosslinker was used instead of TCO-PEG_4_-MAL. SDS-PAGE analysis of the conjugation mixture after reaction and SEC purification indicated that the conjugation yield was comparable to that of the thioether product (AgUox-MAL-HSA) (Figure 4 and Figure 5B). Overall, both AgUox-MAL-HSA and AgUox-APN-HSA conjugates were successfully prepared.

### 3.4. Enzymatic Activity Assays and In Vitro Stability Tests of AgUox-HSA Conjugates

**Figure 6:**
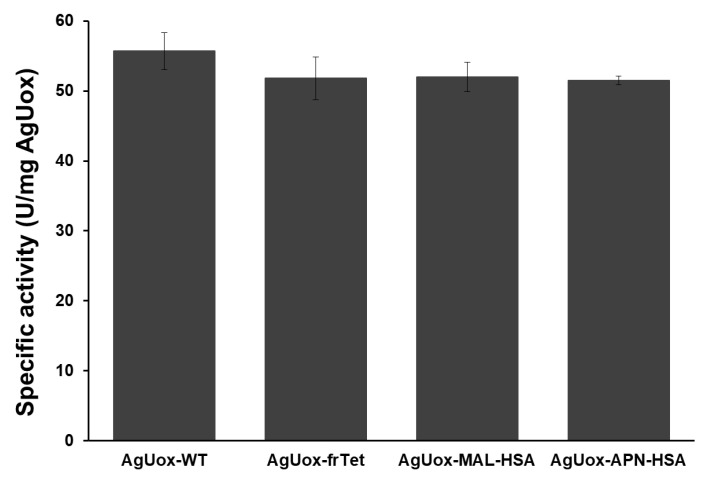
Specific enzymatic activity of AgUox variants (AgUox-WT, AgUox-frTet, AgUox-MAL-HSA and AgUox-APN-HSA) based on mg AgUox. Assays were performed in quadruplicate and error bars indicate standard deviations.

Prior to the in vitro stability tests of AgUox-HSA conjugates, we measured the enzymatic activity of AgUox-WT, AgUox-frTet, and AgUox-HSA conjugates. The enzymatic activity of the AgUox-HSA variants was comparable to that of AgUox-frTet and about 94% of that of AgUox-WT (Figure 6). These results indicate that site-specific HSA conjugation using a crosslinker containing MAL or APN did not substantially reduce the enzymatic activity of AgUox-frTet. The approximately 6% reduction in the enzymatic activity of AgUox-HSA conjugates compared to that of AgUox-WT is likely due to the slight perturbation in the folded structure of AgUox upon frTet incorporation. Despite the slight reduction in the enzymatic activity upon frTet incorporation, the thermostability of AgUox-frTet at 37 °C was similar to that of AgUox-WT after 5 days [36]. Therefore, we believe that comparison of enzyme activity would be an appropriate method for assessing the in vivo stability of AgUox-HSA conjugates.

**Figure 7:**
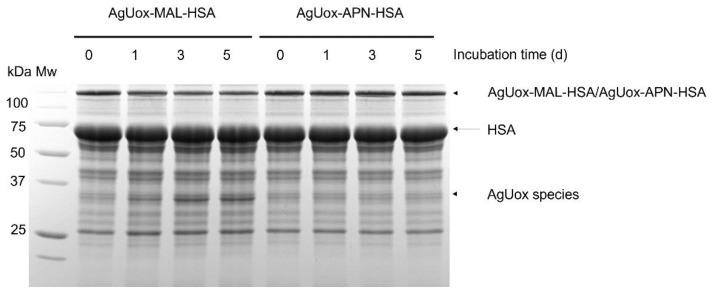
Time-course activity of AgUox-HSA conjugates (AgUox-MAL-HSA and AgUox-APN-HSA) in PBS buffer (pH 7.4) containing 5 μM glutathione and 20 μM HSA at 37 °C. The samples taken at different time points (0, 1, 3, and 5 days) were loaded onto protein gels and then stained with Coomassie Brilliant Blue. Mw: molecular weight standards.

We next assessed the in vitro stability of the AgUox-HSA conjugates in PBS buffer (pH 7.4) containing 5 μM glutathione and 20 μM HSA, mimicking the normal blood environment. The AgUox-MAL-HSA conjugate showed a band corresponding to the AgUox monomer after one- or five-day incubation (Figure 7). To identify the protein in the band, the mixtures were also subjected to MALDI-TOF MS analysis (Figure S1). At the beginning of incubation, two major peaks at 66,489 and 100,372 m/z were observed. They matched well with the expected masses (66,479 and 100,364 m/z) of HSA and AgUox-MAL-HSA, respectively (Figure S1 in Supporting Information). These results also confirm that the monomeric AgUox was conjugated to a single HSA molecule. Since the incubation solution contained HSA, it is not surprising to observe the major peak of HSA. After a one-day incubation, a new peak at 34,208 m/z was observed, which matches well with the expected mass of the cleaved product of AgUox-MAL-HSA (34,192 m/z), the conjugate of AgUox-frTet, TCO-MAL, and GSH (Figure S1 in Supporting Information). This conjugate contains a thioether bond that undergoes retro-conjugate additions and subsequent trapping with endogenous thiols of glutathione; this result is similar to those of previous studies confirming the poor stability of thioether in vitro [30]. In contrast to AgUox-MAL-HSA, neither monomeric AgUox band nor peak of monomeric AgUox was observed in the protein gel or MALDI-TOF MS spectrum after five-day incubation, respectively (Figure 7 and Figure S1 in Supporting Information). These results indicate that the thiol-APN reaction product is more stable in vitro than the thiol-maleimide reaction product under conditions similar to those in blood.

The intact mass of AgUox-WT was 33,310 m/z, which is consistent with the expected mass (33,305 m/z). The mass spectra of the mixture of AgUox-HSA4 variants (AgUox-HSA_MAL and AgUox-HSA_APN) with HSA showed the conjugate and albumin. The masses of AgUox-HSA_MAL and AgUox-HSA_APN conjugates were 100,372 and 100,484 m/z, respectively, which are consistent with their expected masses (100,363 and 100,478 m/z), respectively. The masses of new peaks of the AgUox-HSA_MAL sample after one and five days were 34,208 and 34,206 m/z, respectively, which are comparable to their expected masses when conjugated with AgUox-frTet, TCO-MAL, and GSH (34,193 m/z) with a deviation of less than 0.05%, respectively. AgUox-HSA_APN showed no observable peak of monomeric AgUox.

### 3.5. In Vivo Stability Tests of Uox-HSA Conjugates in Mice

**Figure 8:**
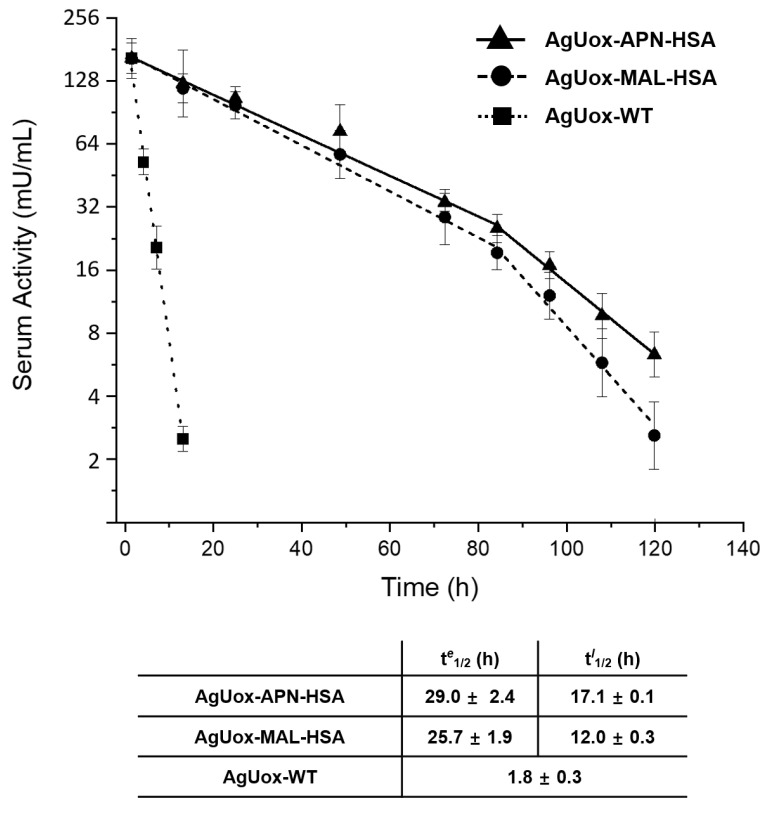
Pharmacokinetic analysis of AgUox-WT and AgUox-HSA conjugates. The serum activity of residual AgUox-WT and AgUox-HSA conjugates was measured in the early phase (0 to 84 h) and late phase (84 to 120 h). t*^e^*_1/2_ and t*^l^*_1/2_ indicate the serum half-lives in the early and late phases, respectively.

To evaluate the in vivo stability of the thiol-APN reaction product, we intravenously administered AgUox-WT and AgUox-HSA conjugates (AgUox-MAL-HSA and AgUox-APN-HSA) to mice (*n* = 4). The half-life of AgUox variants was calculated by measuring the enzymatic activity of serum samples taken at different time points. The serum half-life of AgUox-WT was 1.8 h. The serum half-life of AgUox-HSA conjugates exhibited a two-phase pattern, the early phase (0 to 84 h) and late phase (84 to 120 h). In the early phase, the serum half-lives (t*^e^*_1/2_) of AgUox-MAL-HSA and AgUox-APN-HSA were 25.7 and 29.0 h, respectively (Figure 8). These results indicate that site-specific HSA conjugation with a crosslinker containing MAL or APN substantially increased the serum half-life of AgUox. In the late phase, the serum half-lives (t*^l^*_1/2_) of AgUox-MAL-HSA and AgUox-APN-HSA were 12.0 and 17.1 h, respectively (Figure 8). The reduction in the serum half-life of AgUox-HSA is likely due to the cleavage of HSA in vivo. As expected, the serum half-life of *Ag*Uox-APN-HSA in the late phase was significantly longer than that of AgUox-MAL-HSA. These results indicate that the thiol-APN reaction product is more stable in vivo than the thiol-maleimide reaction product. Although the difference in in vitro stability between AgUox-MAL-HSA and AgUox-APN-HSA was notable, the difference in in vivo stability between them was small. Since each AgUox-MAL-HSA conjugate has four HSA molecules, we speculated that the cleavage of one HSA molecule from AgUox-MAL-HSA did not substantially reduce the serum half-life. Furthermore, during such a long circulation (longer than 84 h), some AgUox molecules can be degraded by proteases in vivo, possibly explaining the smaller difference in in vivo stability between AgUox-MAL-HSA and AgUox-APN-HSA.

## 4. Conclusions

In this study, we investigated whether the thiol-APN reaction product is more stable in vivo than the thiol-maleimide reaction product. For this purpose, the serum half-life of HSA-conjugated AgUox prepared by thiol-maleimide or -APN reaction was evaluated. AgUox-HSA conjugates (AgUox-MAL-HSA and AgUox-APN-HSA) were successfully prepared. Both AgUox-HSA conjugates showed comparable enzymatic activity to AgUox-WT. In vitro stability tests indicated that the thiol-APN reaction product is more stable than the thiol-MAL reaction product-thiol under blood conditions. Pharmacokinetic studies demonstrated that the thiol-APN reaction increased the in vivo stability of HSA-conjugated AgUox compared to the thiol-maleimide reaction, resulting in a longer serum half-life. We believe that the thiol-APN chemistry has great potential for the chemical modification of thiol groups in biomolecules for various biological applications in vivo wherein thiol-maleimide chemistry is used, such as antibody–drug conjugation, antibody–toxin conjugation, and albumin–drug conjugation.

## Data Availability

The supporting data presented in this study are available in Supplementary Materials.

## Supplementary Materials

The following are available online at https://www.mdpi.com/article/10.3390/biomedicines9101334/s1, Figure S1: MALDI-TOF MS spectra of the intact AgUox and the glutathione-treated AgUox-MAL-HSA and AgUox-APN-HSA samples.
