# Supplementary material for: Chemical Modification of Cysteine with 3-Arylpropriolonitrile Improves the In Vivo Stability of Albumin-Conjugated Urate Oxidase Therapeutic Protein
Source: Biomedicines. 2021 Sep 27;9(10):1334. doi: 10.3390/biomedicines9101334 (PMC8533278; doi:10.3390/biomedicines9101334)
Supplement: Supplementary file 1 [file biomedicines-09-01334-s001.zip › biomedicines-1380212-supplementary.pdf]

Supporting Information

Chemical Modification of Cysteine with 3-Arylpropriolonitrile Improves the In Vivo Stability of Albumin-Conjugated Urate Oxidase Therapeutic Protein

Byungseop Yang and Inchan Kwon*

[1] School of Materials Science and Engineering, Gwangju Institute of Science and Technology (GIST), Gwangju 61005, Korea
* Correspondence: e-mail: inchan@gist.ac.kr (I. Kwon); Tel.: +82 62-715-2312

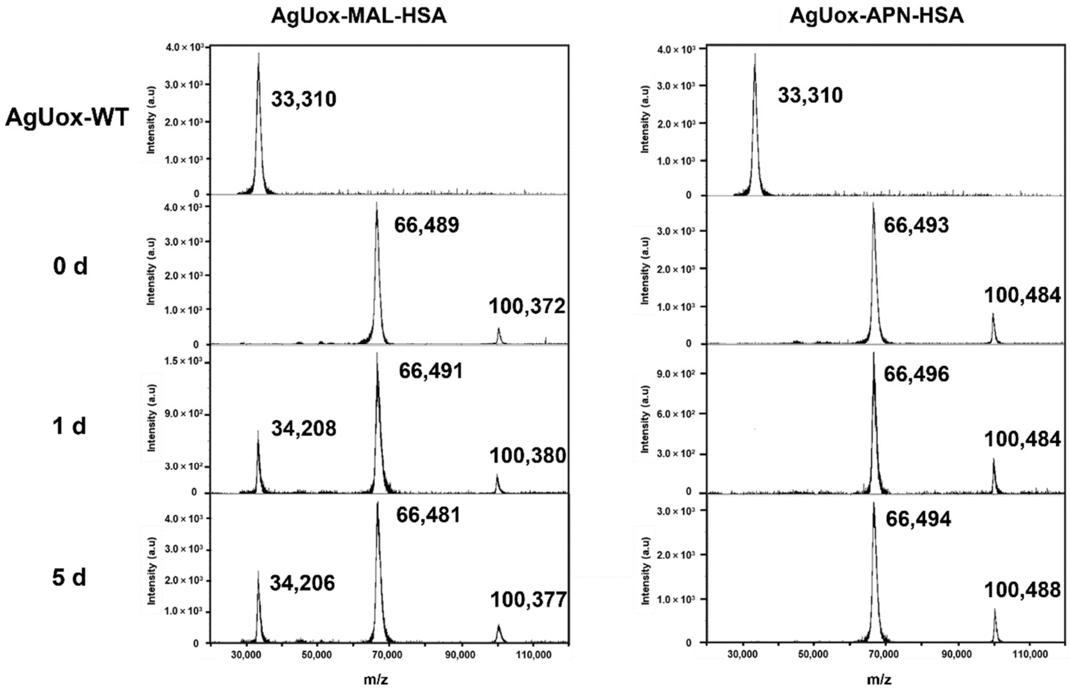

Figure S1. MALDI-TOF MS spectra of the intact AgUox and the gluthathione-treated AgUox-MAL-HSA and AgUox-APN-HSA samples. The samples were mixed with sinapinic acid (SA) matrix solution II (20 mg/mL of SA in 30:70 (v/v) acetonitrile: trifluoroacetic acid 0.1% in water) and then loaded onto target plate coated with SA matrix solution I (20 mg/mL of SA in ethanol). The mass spectra of samples were obtained using a Microflex MALDI-TOF/MS (Bruker Corporation, Billerica, MA, USA).

© 2021 by the authors. Submitted for possible open access publication under the terms and conditions of the Creative Commons Attribution (CC BY) license (http://creativecommons.org/licenses/by/4.0/).
